# Commentary: Leaving tricuspid regurgitation unrepaired at the time of left ventricular assist device implantation?

**DOI:** 10.1016/j.xjon.2020.10.001

**Published:** 2020-10-09

**Authors:** Michael N. D'Ambra

**Affiliations:** Harvard Medical School (ret), University of Maryland School of Medicine, Baltimore, Md

**Figure:**
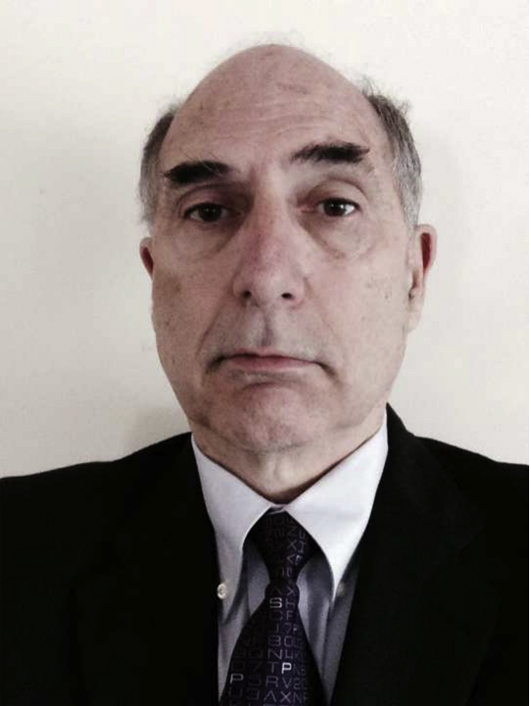
Michael N. D'Ambra, MD

**Central Message**

Leaving TR unrepaired at the time of LVAD implantation?

See Article page 16.

Gomez Hamacher and colleagues describe the impact of tricuspid valve insufficiency on the performance of left ventricular assist devices (LVADs).^1^ They created an ovine model of left heart failure using coronary ligations in 10 animals. The animals were allowed to recover for 2 to 3 weeks, after which they were placed on bypass and underwent LVAD implantation. Five animals also had surgically induced tricuspid regurgitation (TR).

The authors found that in the situation of isolated left heart failure, LVAD performance was not significantly impacted in the TR group, except for a reduction in LVAD outflow relative to the no-TR group. This may be a physiological response of the LVAD to reduced inflow. Sudden imposition of severe TR in a normally functioning right ventricle reduces forward stroke volume as regurgitant flow occurs. Reduced right ventricular (RV) stroke volume translates into reduced pulmonary venous inflow and reduced inflow to the device.

The study question is based on the clinical dilemma of what to do about tricuspid insufficiency when placing an LVAD. Unfortunately, the authors' choice of an left ventricular coronary ligation model and a short recovery period results in pure left ventricular failure with normal RV function. Thus, their study design narrows the utility of the model to answer the study question, because preserved RV function does not often occur in the scenario of LVAD placement. However, there are cases with preserved RV function and TR, and the authors' findings have applicability for these patients.

The impact on LVAD performance is a consideration when contemplating whether to leave an LVAD patient with TR. Other considerations, such as the effects of TR on renal function^2^ and hepatic function,^3^ also must be included in the decision making calculus, as must the potential negative effects of an intervention on the tricuspid valve on repair durability and efficacy.^4^

This remains an important and irksome study question. We look forward to hearing more from these authors in the future.
